# Data management in literature reviews: The C5-DM Framework

**DOI:** 10.1017/rsm.2026.10091

**Published:** 2026-04-17

**Authors:** Gerit Wagner, Julian Prester, Roman Lukyanenko, Guy Paré

**Affiliations:** 1 Management Department, Frankfurt School of Finance & Management, Frankfurt am Main, Germany; 2 The University of Sydney Business School, Australia, Email: julian.prester@sydney.edu.au; 3 McIntire School of Commerce, University of Virginia, USA, Email: romanl@virginia.edu; 4 Department of Information Technologies, HEC Montréal, Canada, Email: guy.pare@hec.ca

**Keywords:** data management, information infrastructure, knowledge synthesis, literature review, research methods

## Abstract

Effective data management is essential for tasks involving decisions based on data, including knowledge synthesis and literature reviews. Despite this, how to carry out data management in literature reviews effectively remains unclear. With the increasing volume of research papers and the expansion of computational techniques for processing data (e.g., machine learning or large language models), it becomes imperative to consider data management as a crucial element for the advancement of literature review practices and tools. Presently, there are shortcomings related to (1) handling the growth of research to be synthesized, (2) addressing data quality issues when applying computational techniques or facilitating the verification of content produced by generative artificial intelligence, (3) enabling efficient reuse of datasets and innovative recombination of tools, and (4) facilitating transparent collaboration across heterogeneous review teams. To address these shortcomings, we develop the C5-DM Framework with conceptual principles to address data management challenges across five areas relevant to literature reviews: data conceptualization, collection, curation, control, and consumption. Methodological guidance for researchers with respect to these five areas is necessary to reduce errors, save time on repetitive tasks, and allow review teams to develop insightful syntheses.

## Highlights

### What is already known?

There is a growing variety in review papers, which involve a range of data management activities. Researchers are challenged to synthesize growing research output, collaborate in teams, utilize advanced computational techniques, and continue to update the review.

### What is new?

We develop a data management perspective for literature reviews and devise the C5-DM Framework with principles to facilitate the flow of data from different sources through the process and toward different forms of outputs. The recommendations are based on the specific characteristics of literature review data, and highlight the distinction of raw and primary metadata, the need to rely on transparent collaboration systems, and the need to select data structures that fit the type of review.

### Potential impact for RSM readers

We advance data management principles for literature reviews, with a focus on methodological pluralism, enabling a systematic approach with transparent reporting, and facilitating the contributions of multiple authors, tools, and computational techniques.

## 1 Introduction

Since data management plays an indispensable role in any endeavor where decisions are based on data, it is a foundation of knowledge synthesis and literature reviews. Yet, the systematic development of data management for literature reviews continues to lag behind, receiving far less attention and methodological guidance compared to the data management fundamentals in empirical, statistical, and machine-learning research.^1^ ^–^ ^3^ This challenge is mirrored by many software packages, which place the burden of data handling, preprocessing, and validation on individual researchers, offering little guidance on how these tasks should be accomplished effectively. It is exacerbated by the increasing volume of research papers utilized in standalone review projects and the expansion of computational techniques for processing data. Literature reviews in most scientific disciplines are becoming big data projects, approaching and exceeding sample sizes of 10,000 papers.^4^ ^,^ ^5^ Such review projects involve having to access, search, retrieve, categorize, and interpret large volumes of data. Consequently, conclusions from literature reviews are directly impacted by the way the requisite data were organized, prepared, and validated.^6^ ^,^ ^7^

Organizing data involves principles for structuring data items and their interrelations, that is, concerns that have been studied extensively in the data management literature.^8^ Considering the redundancies of data retrieved through different search techniques, a key challenge is to design data structures, or schemata, in a way that effectively prevents inconsistencies while preserving the integrity of raw data.

Preparing data refers to managing schema compliance, improving data quality, and preventing error propagation. While methodological data preparation procedures are well-established in statistical data analysis and machine learning,^9^ ^,^ ^10^ there is limited guidance on data preparation procedures in the context of literature reviews. The data creation process for academic publications and their associated metadata is essentially decentralized, often lacking systematic and efficient quality management mechanisms. Errors can be introduced at various stages and may easily propagate through the tools used by review teams, academic search engines, and publishers. These issues are well-known in data management research but are seldom discussed in the context of literature reviews, leaving the outcomes to the varying skills of researchers.

Validating data refers to measures taken to examine the origins of data and to assess their evolution in the process. Examining the origins of literature review data, and the selection of journals or conferences that were covered, is essential to understand potential biases, such as those arising from restrictions to open access papers or the restriction to selected publishers. In the evolution of data, a first concern is to evaluate the completeness and ensure that no paper was accidentally removed, for example, by overly aggressive deduplication procedures. More broadly, many computational techniques, including machine learning and large language models, promise efficiency gains, but also come with imperfect accuracy and varying reliability.^11^ ^,^ ^12^ A responsible conduct of literature reviews should therefore involve the validation of changes introduced by computational techniques,^13^ as well as those introduced by coauthors, research assistants, or even crowdworkers.^14^ ^,^ ^15^

Despite the pivotal role of data management in literature reviews, the organization, preparation, and validation, and their foundational principles remain underexplored, leaving significant gaps in methodological guidelines. Indeed, influential review methods papers^16^ ^,^ ^17^ are silent on how data should be organized to ensure consistency, currency, or correctness when incorporating the varying contributions of researchers and computational techniques. As a result, the absence of well-grounded data management procedures can directly undermine the reliability and validity of review conclusions. From our view, some of the persistent issues in literature review practices and tools can be explained by the paucity of data management principles, and to leverage the ever-expanding data sources and computational techniques in literature reviews, a rigorous foundation of data management is sorely needed.

We introduce the C5-DM Framework to develop the foundations for data management in literature reviews, with data management pertaining to the lifecycle of data, covering the creation, processing, analysis, storage, and archival of data.^18^ We thereby pursue data-centric innovation,^19^ focusing on one of our most fundamental research methods. Developing data management principles in this context fills an important gap, considering that existing principles from other research contexts^1^ ^,^ ^20^ are not immediately applicable to literature reviews.

In the following, we situate data management in the broader context of literature reviews and consider prior research on literature review methodology, along with data management scholarship, to develop the conceptual foundations of literature review data management. Specifically, the data management framework of Chua et al.^21^ informs our work across the areas of data conceptualization, collection, curation, control, and consumption. We adapt and extend prior knowledge on data management to the specific requirements of literature reviews, highlight current shortcomings, and propose guiding principles for data management in literature reviews. We conclude with an outlook on tool development and evaluation.

## 2 Background

### 2.1 Situating data management in the broader context of literature reviews

Methodological work on literature reviews has produced a diverse ecosystem of standards that seek to strengthen review quality, transparency, and reporting. These include widely used reporting guidelines, such as PRISMA 2020 and its extensions,^22^ ENTREQ for qualitative evidence syntheses,^23^ and the RAMESES I and II standards for realist reviews.^24^ ^,^ ^25^ Other frameworks emphasize appraisal and evidence grading, including AMSTAR-2, which evaluates the methodological quality of systematic reviews,^26^ and GRADE, which assesses the certainty of evidence and strength of recommendations.^27^ Finally, search-formulation tools, such as PICOS^28^ and SPIDER,^29^ help structure review questions and inclusion criteria in medical and qualitative research.

Taken together, these frameworks target different phases and genres of review work but share a common emphasis on transparency in reporting and, in some cases, on rigor in the conduct of specific review types. PRISMA and its extensions specify how search strategies, screening decisions, and synthesis procedures should be documented.^22^ ENTREQ and RAMESES tailor this effort to qualitative and realist approaches,^23^ ^–^ ^25^ while AMSTAR-2 and GRADE focus on the quality of reviews and the certainty of evidence they produce.^26^ ^,^ ^27^ Although valuable, these frameworks are largely output-oriented: they prescribe what should be communicated in the final report or how results should be assessed, rather than how data should be organized and managed during the review process.

In parallel, general data stewardship strategies, such as the FAIR Guiding Principles,^20^ promote findability, accessibility, interoperability, and reusability in scientific data. FAIR offers high-level expectations that align with open science priorities, yet it is deliberately agnostic about the structures, formats, and processes specific to different research methods. Consequently, it does not indicate how literature review data should be collected, cleaned, linked, versioned, or transformed, nor how specialized tools should ensure interoperability.

To situate our contribution relative to these initiatives, Table 1 compares major frameworks in terms of their purposes, scopes, and regulatory emphases, and highlights the gaps they leave regarding data structuring, interoperability, versioning, and reuse. This mapping shows that although existing frameworks provide essential guidance on reporting, appraisal, and evidence grading, none offer a comprehensive approach to the management of review data across its lifecycle.

**Table 1:** Comparison of major frameworks relevant to literature reviews Table 1 Long description.

			What it does not
Framework	Primary purpose	What it provides	provide
PRISMA 2020 and extensions^22^	Reporting of systematic reviews	Standards for reporting search, screening, and synthesis	No guidance on data structures, versioning, or workflows
ENTREQ^23^	Reporting of qualitative syntheses	Standards for reporting qualitative methods and synthesis	No guidance on coding files, extraction structure, and traceability
RAMESES I and II^24^ ^,^ ^25^	Method and reporting of realist reviews	Protocol, synthesis logic, and reporting standards	No data formats, versioning, or interoperability
AMSTAR I and 2^26^	Critical appraisal of systematic reviews	Criteria to evaluate methodological rigor	No guidance on data preparation, screening workflows, and schemata
GRADE^27^	Evaluation of certainty of evidence	Rules for rating evidence certainty	No data formats, extraction structures, or literature review processes
FAIR^20^	Principles for scientific data stewardship	Principles related to findability, accessibility, interoperability, and reusability	No literature review-specific schemata or data lifecycle guidance
PICOS/SPIDER^28^ ^,^ ^29^	Structure for review questions and search strategies	Templates for defining inclusion criteria and creating search strategy building blocks	No guidance on data inputs, logs, extraction, or transformations
C5-DM (this article)	Data lifecycle management for literature reviews	Principles and practical recommendations for managing literature review data (the C5-DM Framework)	Does not replace standards for reporting or evidence assessment

This gap is consequential. No current guideline defines what constitutes literature review data or how such data should be handled from end to end. Reporting standards focus on narrative transparency, and data stewardship principles focus on general accessibility, but neither addresses the data foundations of review practice—namely, the preparation, structuring, validation, transformation, versioning, and reuse of the diverse data objects produced as a review unfolds. These include raw and cleaned metadata, screening decisions, coding files, extraction tables, full-text documents, synthesis artifacts, and audit logs, none of which are governed by existing frameworks.

As a result, review workflows often rely on fragmented toolchains, proprietary formats, bespoke spreadsheets, and undocumented data transformations. Such practices hinder reproducibility, limit traceability, and reduce opportunities for reuse, especially as reviews scale in size and complexity or incorporate computational tools.

Our framework addresses this gap by proposing a data lifecycle perspective that conceptualizes literature reviews as data-intensive, multi-stage processes encompassing five areas: conceptualize, collect, curate, control, and consume. This perspective clarifies what literature review data is, how it evolves, and how it should be structured, validated, transformed, and versioned. In doing so, it complements established reporting and appraisal standards while supplying a missing foundation for coherent, transparent, and interoperable data management in literature review projects.

For ease of reference, we label our framework C5-DM, denoting a five-stage data lifecycle approach to literature review practice: conceptualize, collect, curate, control, and consume. The acronym foregrounds our central claim that systematic data management is not a peripheral concern but a foundational condition for transparent, reproducible, and scalable review science. Accordingly, C5-DM serves as a unifying heuristic for understanding, designing, and evaluating data workflows in literature reviews.

### 2.2 Foundations of data management

Prior research on data management builds on a long-standing tradition in disciplines, such as computer science, information systems, and information retrieval. According to Chua et al., data management is “concerned with activities and methods to conceptualize, collect, curate, consume and control data.”^21^ From a lifecycle perspective, this involves the creation, processing, analysis, storage, archival, and destruction of data.^18^ Across these generic stages, data management practices must be adapted to the nature and evolution of the data involved. This includes the involvement of different stakeholders, the standardization of procedures and architectures to avoid fragmentation, and the controlled flow of data across organizational boundaries.^30^

From the range of data management frameworks available,^18^ ^,^ ^30^ ^,^ ^31^ we adopt a recent one, which was proposed in the context of *MIS Quarterly* research curations.^21^ This framework synthesized relevant knowledge from the IS discipline’s top journals, along with more recent thinking and advances in data management research and practice, to propose general activities of data management. Its generality, parsimony, and compatibility with other data management frameworks^32^ make it an appropriate foundation for data management in literature reviews. Prior work has broadly formed research streams on five interrelated areas of data management.

The five interrelated data management areas are conceptualization, collection, curation, control, and consumption.^21^ Conceptualization involves analyzing and representing data requirements and domain knowledge through informal or formal models (e.g., entity-relationship diagrams), guiding subsequent management activities. The collection focuses on identifying data sources, designing extraction and integration protocols, and ensuring data quality, evolving from intra-organizational data handling to crowdsourced and artificial intelligence (AI)-driven techniques. Curation involves the development of infrastructures that store, index, and manage data for efficient access, while integrating versioning, quality control, and sustainability considerations. Control establishes governance policies, security measures, and data structure standardization to protect, regulate, and optimize data utility within organizational and technical frameworks. Finally, consumption applies data management principles to analyze, transform, and repurpose structured and unstructured data for decision-making, insights, and analytics, increasingly leveraging ML, big data processing, and scalable architectures. Together, these areas form a comprehensive approach to effective data management.

## 3 The C5-DM Framework for data management in literature reviews

**Figure 1:**
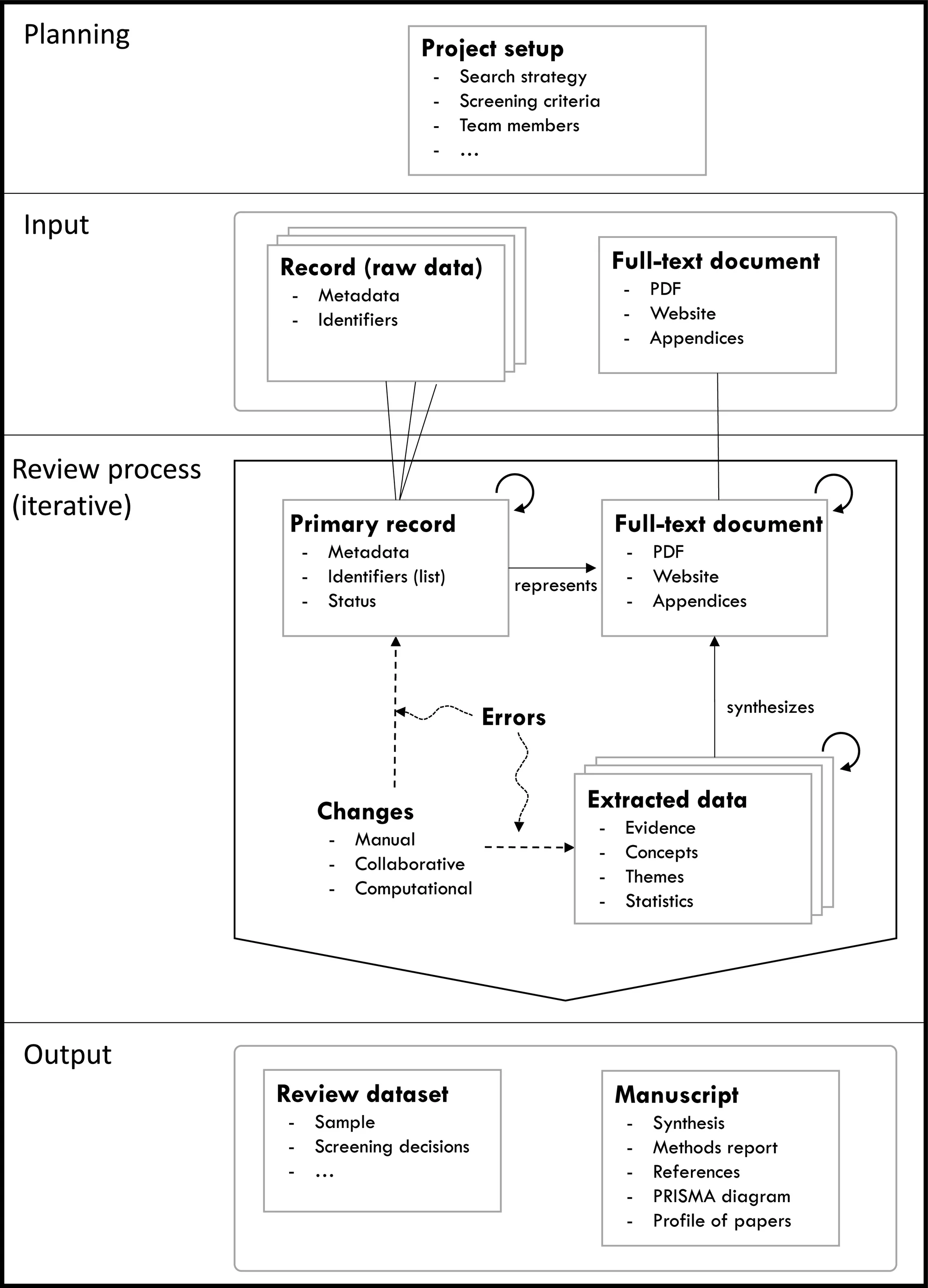
A conceptual model of data in literature reviews. Figure 1 Long description.

We now build on prior data management work^21^ to develop the C5-DM Framework for the literature review context (see Table 2). In doing so, we adopt a comprehensive view, covering data related to the project setup, input, process, and output (as illustrated in Figure 1). We use the five areas—conceptualization, collection, curation, control, and consumption—as a foundation to discuss the effective management of data in literature reviews. For each area, we summarize the state of the art and current limitations from the perspective of each data management area before developing principles to address these challenges and formulate specific recommendations for prospective authors. At the end of the section, we present an online vignette that offers practical illustrations and resources for each recommendation.

**Table 2:** The C5-DM Framework: Areas, principles, and recommendations Table 2 Long description.

Areas and principles	Data management recommendations^b^
**I. Conceptualize** A comprehensive view of literature review data covers input, process, and output, together with the project setup, as illustrated in Figure 1. Literature review data are characterized by dynamics, non-determinism, and atomicity.	Store search results separately from the primary data, using identifiers as links. Treat manual data work as primary analytical procedures by documenting parameters and tracing changes.
**II. Collect** A definition of metadata quality must be stated and automated data preparation procedures are required. API-based searches enable automated and standardized retrieval, whereas graphical database interfaces introduce friction due to manual operations, format conversions, and potential errors.	Document database coverage, especially when using LLM-based tools. Set up data preparation procedures to ensure high-quality and machine-readable data.
**III. Curate** Transparent data management systems with a history of the literature review project are essential for reliable collaboration. Online repositories support sharing and reuse of data, while local repositories offer cheaper storage and computation. Data structures and tools must fit with the nature of the data extraction, analysis, and philosophy associated with the selected type of review.	Rely on a version-controlled repository to organize and trace review data. Align data structures with the specific review type.
**IV. Control** Standardized data structures are a precondition for inter-operability of tools and scalable collaboration. Data control involves measures to ensure confidentiality, integrity, and availability. Data quality management requires measuring and tracing errors, and, ideally, correcting them at source.	Select standard file formats to facilitate access and contribution by humans and software. Adopt non-redundant data structures, supported by identifiers and automated validation procedures. Assess all manual and computational changes and roll back unreliable contributions.
**V. Consume** Reuse refers to the use of record metadata, screening decisions, or extracted data in follow-up research. Reuse of literature review data may occur manually or computationally.	Reuse data from prior literature review papers and curated repositories. Share review data via open data platforms and use open licenses to enable reuse.^a^

a For guidance on selecting open licenses, see https://choosealicense.com/.

b Illustrations and further resources are available in the online vignette (https://fs-ise.github.io/C5-DM-vignette/), see Figure 4.

### 3.1 Conceptualize: Defining and characterizing literature review data

#### 3.1.1 State of the art and limitations

In contrast to the established practices of data management in many research methods areas, in the literature review context, conceptualization has not seen much attention. Literature review methods papers tend to focus on the level of knowledge, synthesis, and interpretation,^16^ ^,^ ^33^ and have heretofore neglected conceptual foundations and guidelines focused on data. Notable exceptions can be found in the context of data extraction and analysis for meta-analyses,^34^ which is limited to a particular type of review. In addition, prior research has proposed relational data models for literature reviews, most of which are restricted in scope.^35^ ^–^ ^37^ As a result, there is currently no comprehensive definition for “data” in the context of literature reviews.

Efforts to conceptualize literature review data have focused on specific aspects, leaving a number of gaps. Prior research has advanced conceptual foundations of data for search queries,^38^ in inductive hierarchical coding,^39^ deductive tabular analyses, also known as concept matrices.^16^ Further work has offered conceptual ontologies to characterize relations between papers.^40^

Despite serving the same method, these contributions are not integrated into an overarching conceptual view of literature review data, and they raise the question whether there may be other conceptual modeling approaches better suited for representing literature review data. In particular, to conceptualize means to understand how the original data were collected, answering questions like (1) Who created the data? (2) Why was the data created? (3) When and where was the data created and altered? or (4) By what means was the data created? In addition, some data in the literature review context are already pre-collected—by the authors of the source papers—raising the need to first and foremost understand data from the original studies. Prior work indicates that it is increasingly critical to know who created data and what motivated the creator.^41^ ^,^ ^42^ This can aid in assessing source credibility and detecting biases, as well as assessing the accuracy and completeness of data. For example, it is evident that paper metadata is handled differently by publishers or by third-party metasearch engines like *Google Scholar*. Similarly, understanding whether a paper was published in a predatory journal, or whether it was retracted or corrected, is important when assessing the quality of evidence or running subgroup analyses.

#### 3.1.2 Principles

To advance data conceptualization, we propose broadening the conceptual framework for literature review data and recognizing its specific characteristics (as illustrated in Figure 1). First, we suggest expanding the conceptualization of data in the literature review beyond traditional record metadata. As a foundation, we define literature review data broadly as encompassing all data collected and stored about:

1. the papers in the sample, including the record metadata (raw and primary), the full-text documents, and complementary materials, such as appendices, datasets, post-publication peer reviews, and social media mentions;
2. the review process, including details related to the search, the screening decisions, and data recorded as part of the extraction or synthesis steps;
3. the review project, including the review objectives, methodological choices, the team, and the schedule.

With this conceptualization, we intentionally propose a comprehensive view of literature review data. Specifically, we adopt a process-oriented perspective that encompasses raw input data, data capturing the progression of records through various stages of the review process, and the different output formats generated. This data is documented within the context of a review project, ideally maintained in a versioned history, and generated through manual, collaborative, or computational procedures. While this comprehensive conceptualization may appear obvious, current efforts in this area often adopt a more limited perspective. For instance, review data curated as part of the *SYNERGY* dataset^1^ are restricted to record identifiers and pre-screening decisions.^5^ Similarly, the view of review data developed by Haddaway et al.^43^ focuses on extracted data exclusively.

Second, the nature of data should be conceptualized appropriately. In this regard, we contend that literature review data have distinct characteristics that differ from data processed by other research methods and require a distinct data management approach:

- *Dynamics*: In literature reviews, data are in motion, meaning that input metadata as well as interpretations may evolve dynamically throughout the process. For instance, Leidner and Tona^44^ emphasize that theorizing, in the data synthesis step of a review and theory development paper, can be highly iterative with compositions evolving through multiple steps. Notions of immutable raw input data commonly adopted for reproducible computational analyses^2^ are insufficient.
- *Non-determinism*: In literature reviews, processing operations are often manual and inherently non-deterministic. As such, only parts of the processes that introduce changes to the data are repeatable in the sense of reproducible research analyses that are codified and reliably produce identical results upon repeated execution. Common notions of deterministic computational processing operations^45^ do not apply.^46^
- *Atomicity*: In review projects, processing occurs on a per-paper basis, individual decisions must be recorded, and validation, as well as potential corrections on a more granular level, are required. The notion of processing the dataset as a whole is insufficient, such as the common approach of performing matrix operations on the whole dataset.^3^

In essence, conceptualizing literature review data appropriately requires awareness of the activities of structuring, tracking, and updating data in a review project. With the following recommendations, we caution against the use of general research data management approaches or reproducible research frameworks.^45^ As discussed previously, these frameworks do not fully align with the inherent characteristics of literature review data.

#### 3.1.3 Recommendations

**Figure 2:**
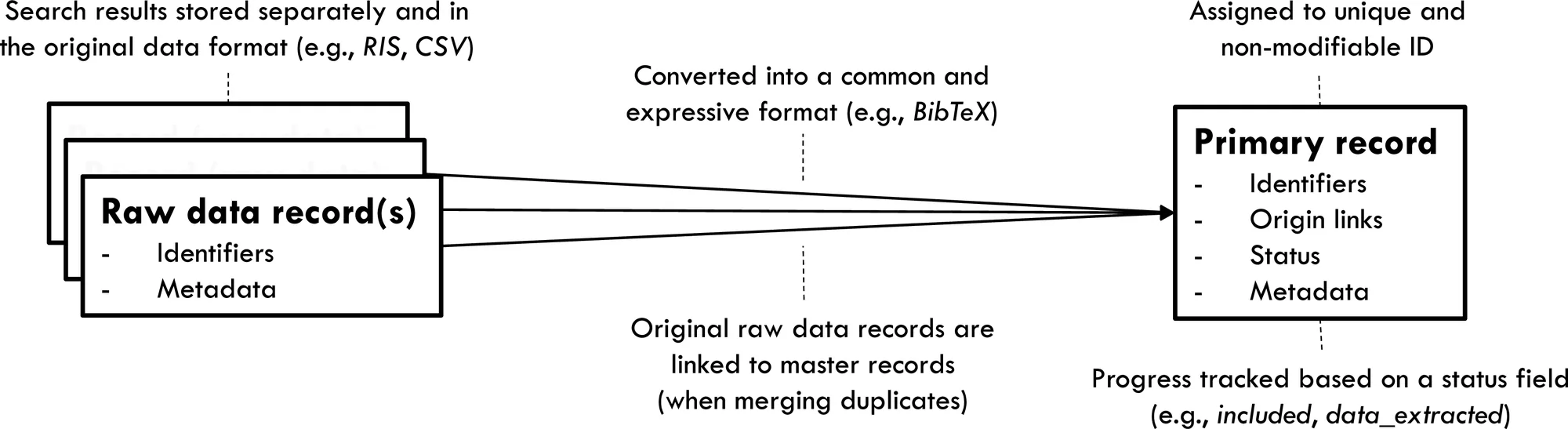
Illustration of the metadata management recommendation. Figure 2 Long description.

*Store search results separately from the primary data, using identifiers as links:* Search results retrieved from academic databases, as a form of raw data, should be preserved in their original, immutable form, while primary data may be modified manually or computationally. To connect them, identifiers should be used to link each primary record to its raw sources (see Figure 2). This separation is essential for both methodological and legal reasons. From a methodological perspective, data management in literature reviews differs from empirical and statistical research: primary review data are not immutable but evolve through iterative human and computational changes. Maintaining immutable raw records alongside mutable status and linkage structures enables traceable and auditable deduplication, while eliminating the need to replicate primary record status across multiple raw data entries. From a legal perspective, licensing restrictions often prohibit sharing raw search results obtained from commercial databases, whereas sharing identifiers and derived primary data often remains possible and necessary for reporting purposes.

*Treat manual data work as primary analytical procedures by documenting parameters and tracing changes:* In literature reviews, core steps, such as screening, deduplication, data extraction, and synthesis, are typically performed through manual or semi-automated procedures that fundamentally shape the review dataset and its conclusions. Instead of taking the form of informal and undocumented activity, manual procedures should therefore be documented, versioned, and traceable in the same way as computational ones. For each task, review teams should specify a trigger (when the task is performed), the responsible person(s), the tools and commands used, how changes and outcomes are recorded, and the validation steps applied before or after the changes are accepted. Capturing this information in a version-controlled history enables collaborators to inspect, validate, and reverse individual contributions, while also providing an auditable record for reporting and reuse.

### 3.2 Collect: Collecting data in literature reviews

#### 3.2.1 State of the art and limitations

In the context of literature reviews, collection involves data sources as well as the procedures to retrieve and prepare metadata and full-text documents for the screen and analysis. It is instructive to remember that the data production and retrieval process crosses the boundaries of multiple organizations and technical systems, including the authors of primary papers, the peer review system, the publishers’ manuscript production and publication facilities, the institutions registering metadata and issuing identifiers (e.g., *Crossref*/DOI), academic database indices, metasearch engines, scholarly networks, reference management systems, and literature review tools. In addition, the retrieval of metadata and full-text documents is complicated by paywalls and different subscription models for academic databases and publisher content.

The quality of collected data varies considerably, calling for a clear understanding of metadata quality and more effective tool support for (meta) data provenance. Defects in (meta) data quality are inherent in a highly distributed data generation process, which involves a range of independent actors (authors, editors, proofreaders, publishers, and database providers) and manuscript production pipelines, which may even predate the digital era. Yet, data quality defects are hard to measure and are rarely fixed at source. Especially in some of the emerging AI-based databases and literature review tools, sustainable approaches to data quality provenance do not seem to be a priority.^3^ In addition, each member of a review team may retrieve different results from databases and have access to different subsets of the full-text documents.^38^ As such, academia presents a decentralized data management setting of extraordinary complexity. Prior data management research offers context-reflective models for data quality defects and resolution.^49^ This work sensitized practitioners to the error rates of manual data processing, especially in settings with rapid growth of data and incompatible systems, and developed a nuanced conception of data quality dimensions.^50^

With data collection procedures in literature review projects typically involving multiple systems, data handling, and format conversion are often completed in an ad-hoc manner without a reproducible standard for structuring, processing, and reporting the data collection.^51^ Academic databases, as the primary source of data collection in literature reviews,^52^ continue to restrict effective searches, slowly implement new functionalities, and contain several bugs.^53^ Specifically, many databases require users to operate web-based interfaces, which can be error-prone,^53^ hard to reproduce, and particularly inefficient when updating literature searches. Some providers have started to offer application programming interfaces (APIs), but integration into literature review tools is limited, possibly because nested Boolean queries are not always supported, query complexity is restricted to save computational efforts, and the number of results is limited.

#### 3.2.2 Principles

As a first and foundational principle for data collection, a definition of metadata quality is required, along with corresponding data preparation procedures. The challenge is that publications do not have a singular *real-world* entity based on which the correctness of metadata could be assessed.^54^ Metadata registered with the DOI can change over time, not all publications are registered, and the data deposited on publisher websites or in PDF documents may also differ. This is underlined by the APA manual,^55^ which specifies many rules for the formatting of citations, that is, representational consistency, but does not provide any guidance on what should be considered the correct metadata value. Given that there are no absolute definitions of correct metadata, and that metadata may not necessarily be consistent across sources (PDF, *Crossref*, and publisher websites), researchers may primarily rely on heuristic rules. One approach would be to consider metadata correct when they are up-to-date and identical across different sources. In case of disagreements, only one version can be considered correct. Ideally, the decision of which version is considered correct is made by the publisher, the authors of the original paper, the community, or individual researchers. In practice, few if any tools assess the consistency and correctness of metadata, given that additional effort is required, and that organizations and individuals like publishers or authors may not be available. Furthermore, quality assessment and preparation of data are essential. For records, this would involve measuring the completeness of metadata and allocating preparation efforts accordingly, primarily to prevent errors in duplicate removal.^56^ Input data can have a variety of quality defects, and manual, collaborative, and computational changes are introduced with varying reliability. Given that literature review steps build on each other in multiple iterations, careful data handling is needed to prevent the propagation of errors, which may ultimately affect the conclusions of the review. A related task is to identify retracted studies or errata notes. In addition, keeping metadata up-to-date can be essential to make sure that retracted papers are handled appropriately. Finally, ensuring quality and machine-readability is an essential facet of quality management for full-text documents.

Second, leveraging APIs for retrieval can help automate and standardize collection methods, which is necessary for managing the increasing volume and variety of literature review data. Currently, literature searches are primarily conducted manually, through the graphical web interfaces of database providers, introducing inefficiencies due to manual operations, format conversions, and the potential for errors. Similar to ETL pipelines used in data warehousing, the variety of database interfaces and search techniques becomes more manageable when accessed through standardized wrapper methods or programmatic API integration. Search queries would benefit from a standardized and validated syntax, as highlighted by Li and Rainer.^53^ Moreover, the standardization of data schemata for search results, drawing on schema integration techniques,^57^ is rarely implemented in current literature review tools. Clearly defined record identifiers across databases are essential for enabling efficient, highly automated search updates, as envisioned in prior work.^58^

#### 3.2.3 Recommendations

*Document database coverage, especially when using LLM-based tools:* Transparent reporting on data access is critical to ensure the comprehensiveness and reproducibility of literature reviews. Researchers should systematically analyze the coverage of journals, conferences, and grey literature, including the availability of search fields (titles vs. abstracts), and embargo periods. When new LLM-based tools,^4^ such as *Elicit* and *Consensus*, are used, it should be explained how paywalled content was accessed, given that such tools are often limited to openly accessible data.

*Set up data preparation procedures to ensure high-quality and machine-readable data:* It is crucial to implement data preparation procedures that ensure reliability and accuracy, especially when relying on large-scale AI-based search tools like *Semantic Scholar*. Key dimensions include metadata completeness and correctness,^50^ and mechanisms for identifying retracted papers or predatory journals. Prioritizing tools that support high-quality data preparation and machine-readability will contribute to the validity of literature review outcomes.

### 3.3 Curate: Infrastructures for literature review data

#### 3.3.1 State of the art and limitations

In curating literature review data, there is limited guidance on how literature review methodology, including the growing variety of goals and types of reviews^33^ ^,^ ^59^ translates into requirements related to the structure and quality of data. A plethora of guidelines and tools focus on systematic reviews and meta-analyses, often specific to the medical sciences. Yet, the epistemological and methodological assumptions, along with the review procedures and data structures, are only appropriate for selected types of reviews. For inductive work, the Gioia data structure^39^ is well established but also restricts users to the modeling of single-hierarchy concepts, themes, and dimensions. For scientometric work, there are new challenges for analyses that use content to enrich structural models created from the metadata of publications.^60^ For conceptual work, recent developments go beyond concept matrices, as suggested by Webster and Watson,^16^ and point to the potential benefits of multi-level knowledge graphs representing concepts and concept relationships.^61^ In the absence of an overarching framework, data curation activities often occur on an ad-hoc basis and may not fit well with the specific requirements of the respective review type.

Current tools supporting the curation of literature review data fall into two major categories: workflow solutions and specialized applications. Workflow solutions, such as the *Covidence* platform, are typically designed to cover the whole review process. In workflow solutions, data curation challenges include limited data interoperability, lack of programmatic interfaces, and lock-in effects, which are common in proprietary data ecosystems.^62^ In addition, it is evident that many of these platforms operate on relational databases, which may not be the most suitable choice for editing semi-structured qualitative and quantitative data in teams and with computational techniques, which vary in performance.^5^

The second category of specialized applications includes tools like reference managers, spreadsheet software, and AI-based tools.^63^ ^,^ ^64^ Tools in this category often require researchers to perform format conversion and keep track of the sample, that is, the status of each record in the process. Despite the plethora of review tools available, there are major limitations related to the curation of review data. Many tools, especially workflow solutions, restrict data accessibility, require researchers to perform error-prone format conversions, and effectively restrict the interoperability of data curation tools.^65^ In addition, current tools generally offer limited transparency of the different changes applied to a review dataset. While data curation problems are harder to diagnose with proprietary tools, it is evident that few reliable, open-data tools are available for curating literature review data.

#### 3.3.2 Principles

First, selecting a transparent data management system is essential for reliable and effective collaboration, involving researchers and computational techniques. While many (web-based) literature review tools build on relational databases, we are not aware of tools that build on collaborative versioning systems like *Git*.^6^ This is surprising given that research data management for other methods heavily relies on *Git*,^67^ ^,^ ^68^ and considering the specific benefits of using *Git* for managing collaborative work on textual data. Both systems differ fundamentally with regard to the transparency of change sets and capabilities to analyze and undo changes. In the context of literature reviews, in which researchers need to collaborate with different contributors and computational techniques, the capabilities of data management systems may lead them to adopt different approaches. If changes are not immediately transparent and hard to undo, it is reasonable to adopt a “protective boundary approach,” in which researchers restrict access and categorically prevent contributions from many potential contributors and tools. However, if changes are transparent and easy to undo, researchers may adopt a “test–validate–undo approach,” in which contributions from different contributors and new tools are solicited, evaluated, and accepted or undone. As such, operating with the latter approach and collaborative versioning systems is essential for adopting innovative tools, such as those based on AI,^63^ ^,^ ^64^ work in larger teams,^69^ or include crowdworkers.^14^ Going beyond relational databases and exploring the opportunities of *Git*-based literature reviews can therefore be seen as an essential but neglected precondition to developing more efficient, reliable, and innovative literature reviews.

**Figure 3:**
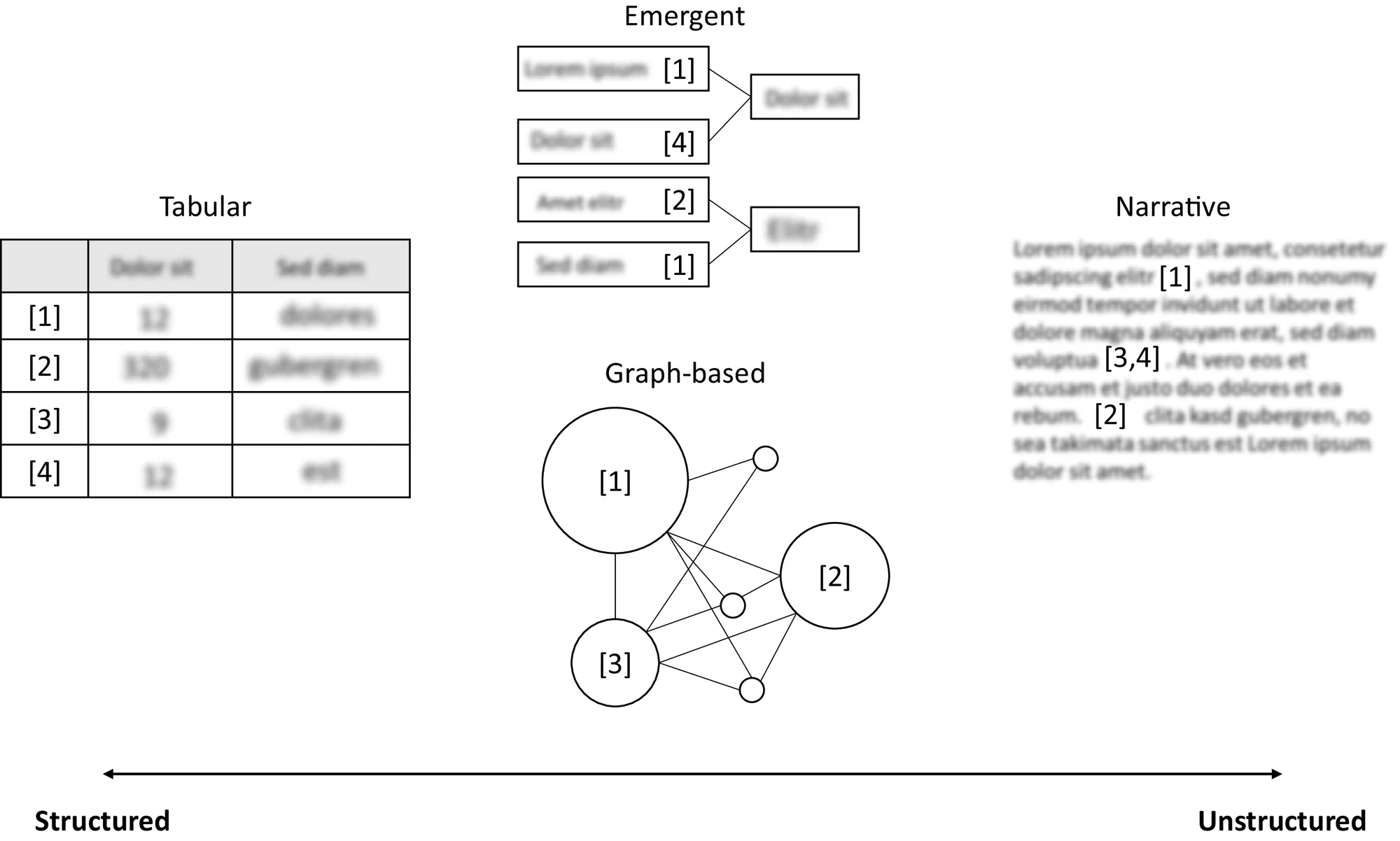
Plurality of structures in literature review data. Figure 3 Long description.

Second, it is essential to align data structures with the requirements of the review type. As illustrated in Figure 3, literature reviews can involve various forms of structured, semi-structured, or unstructured data. For instance, meta-analyses often involve highly structured data that is tabulated in deductive procedures. Theoretical reviews can adapt interpretive and inductive approaches, relying on semi-structured forms of emergent data coding, as exemplified by hierarchical Gioia data structures^70^ or heterarchical, graph-based approaches.^71^ Finally, unstructured forms of review data occur in the synthesis section of every standalone review paper, with narrative reviews typically relying on this form of data exclusively.^7^ Our key argument here is that data structures can be highly specific to review types, and that the most significant differences lie in the data extraction and analysis, as opposed to the search, deduplication, or screening steps. This has fundamental implications, which are yet to be fully appreciated:

- First, there is a need for review tools supporting different review types and data structures *in the data extraction and analysis stages*. The excessive use of systematic review approaches and tools for other review types has been criticized repeatedly.^74^ In fact, the use of highly structured tabular data forms for emergent or interpretive syntheses can be seen as a prime example of the “law of the instrument,” underscoring the need for a differentiated arsenal of tools that fit the respective review type.
- Second, there is a *limited or non-existent* need to use or develop review-type-specific tools for the early stages of the review process. In these stages, the structure of data can even be standardized without the need to differentiate between the many review types that have been proposed (e.g., Amog et al. list 41 different types of reviews^75^). In fact, the challenges of handling data properly in the early stages, for example, in duplicate resolution or syntactical search query validation, involve their own highly specific challenges and are hard to address effectively.
- Third, mechanisms to synchronize the sample need to be adapted to the respective data structures. For instance, when searches are updated or when more restrictive screening criteria are applied to remove papers from the sample, the synchronization mechanisms differ between the data structures illustrated in Figure 3. In tabular data, it may often be possible to specify how new studies are to be added or removed. In semi-structured data, identifying the elements and relationships can be helpful. Finally, in unstructured narrative syntheses, the interpretive analysis of researchers needs to be triggered.

In sum, the implications highlight the need to carefully select appropriate data structures, and to align the mechanisms for synchronizing the sample accordingly.

Third, the advantages of local and online deployment of data repositories and tools must be considered carefully. In particular, common server- and web-based deployment models should be reconsidered, given that they come with a number of inherent restrictions. Surprisingly, this category of deployment is the most common one among ML-based literature review tools.^76^ The cost of computational resources, for example, for duplicate identification or analyses of full-text documents, is a restriction, considering the growing volume of literature and expectations for review projects. While some researchers may be able to afford costly subscriptions for commercial platforms, many others may need to rely on local computational resources, which are more readily available. In addition, restrictions related to copyrighted full-text documents and individual subscriptions for retrieval services (APIs) are typically granted to individuals (through their institution), giving local environments a unique advantage over web-based environments in which all documents and subscriptions must be entered individually. Overall, current systems primarily offer user-friendly proprietary interfaces,^76^ but are less suitable for programmatic computational access, innovation, or (continuous) evaluation.

#### 3.3.3 Recommendations

*Rely on a version-controlled repository to organize and trace review data:* We recommend using a version-controlled repository to curate literature review data, including record data, full-text documents, extracted data, and outputs. To accomplish this, *Git* provides a good fit, as it enables tracking changes, evaluating contributions, and maintaining a transparent revision history. In addition, it offers powerful features for collaborative reviews in which changes from different contributors must be reviewed and integrated. The use of collaborative versioning systems is an established element of research data management,^68^ with tutorials available online. In the context of literature reviews, it can be particularly instructive to familiarize oneself with example projects, such as the review of COVID-19 research.^66^ In addition, distributed version control systems allow researchers to synchronize local and online repositories, offering a convenient way to benefit from local computational resources while making the data available for reuse.

*Align data structures with the specific review type:* Different types of reviews come with different expectations regarding their outputs, and how the data are handled and structured. For example, systematic reviews rely on study-centric evidence extraction and tabular summaries; bibliometric reviews focus on key publications and the evolution of knowledge over time to reveal intellectual structures; and theoretical reviews involve inductive coding of themes across papers to support higher-level abstraction. These differences require review-specific data structures and, consequently, extensible underlying schemata that can accommodate evolving requirements, as exemplified by standards such as Darwin Core. Authors should therefore explicitly define the data structures required by their focal review type before evaluating tools. When selecting tools, it is essential to critically assess whether developers’ claims about supported review types reflect true alignment with the required data structures, instead of accepting method–tool misalignment driven by the limitations of a given platform.

### 3.4 Control: Provenance of literature review data

#### 3.4.1 State of the art and limitations

Controls should ensure that data management practices neither bias the conclusions of a literature review nor hinder the efficiency or innovativeness of the review process. In addition, control is critical for ensuring that data are handled ethically and responsibly. Review conclusions can become artifactual, that is, driven by methodological errors rather than reflecting true relationships, when input data contain quality defects or when manual or machine-generated content introduces errors that go uncorrected. Our focus, therefore, is on implementing quality management measures for input data and examining the interplay of data transparency, evaluation, and correction. When handling (meta) data retrieved from academic databases or PDFs, it is essential that data curation systems effectively trace both the original inputs, whose quality may vary, as well as the changes introduced through manual or computational quality improvement processes.

In the context of literature reviews, several challenges emerge: human annotation, data extraction, and classification tasks often exhibit imperfect reliability^77^; manual data entry in large datasets is prone to errors^77^; and computational techniques designed to analyze semi-structured data are frequently probabilistic, meaning they are not perfectly accurate and heavily depend on the quality of the input data. As van de Schoot et al.^78^ highlight in the documentation of *ASReview*, this leads to the well-known “garbage in, garbage out” problem. Importantly, these issues can compound and reinforce one another across the iterative stages of the review process, amplifying potential bias if not properly managed.

With regard to transparency,^8^ evaluation, and correction, curation tools should support the tracing of data throughout the review process, that is, from raw data sources, their matching to primary records, to the extracted data, and the outputs of the review. The issue of data provenance has been researched in the context of general databases,^80^ and in the context of scientific workflows.^81^ Yet, we are not aware of existing tools that transparently track changes in the context of review projects. Overall, data traceability and transparency figure as key limitations of tools curating literature review data, requiring researchers to rely on manual and inefficient procedures to evaluate changes and make corrections.

In addition, voluntary control of data structures is an essential element to facilitate efficient data management and a combination of different tool innovations. This is underscored by foundational work in the context of statistical software in R,^3^ or standardization efforts for machine-learning libraries (e.g., the *ivy* framework). When tools are based on a shared data structure, considerable efforts for data conversion and manipulation can be saved. Although promising initiatives have started in the context of literature reviews,^43^ different steps of the review process and various review types remain to be considered.

#### 3.4.2 Principles

While control applies to the whole literature review project, including the planning and output stages, we focus on control in the data management activities of the review process. First, we propose that the adoption of shared data structures is essential as a form of voluntary self-regulation in literature reviews, aligning with standardization efforts seen in fields, such as statistical analyses or ML. There is a clear need for a comprehensive data structure that spans all steps of the literature review process—covering everything from search to synthesis—while accommodating both semi-structured and unstructured data. This structure should not be limited to the synthesis step but should extend across the entire review workflow, clearly distinguishing between raw data and integrated findings. Given that individual researchers have the autonomy to define their own data structures, the lack of standardization can lead to inconsistencies that affect the reproducibility and reliability of review outcomes. Existing approaches, such as those seen in the *SYNERGY* datasets, often face challenges related to data quality and redundant storage, which can result in inconsistent results. Moving toward a de facto standard, similar to the one represented by the *tidyverse* package in statistics, would significantly enhance consistency, promote data integrity, and reduce errors across literature review projects. Figures 1–3 may serve as an initial framework to support this standardization effort.

Second, measuring and controlling data quality is crucial throughout the literature review process. This involves labeling data for quality defects, ensuring data integrity, and maintaining a clear trail of changes to capture who made specific alterations. Auditability of records should be ensured at every step, allowing for transparent and responsible oversight of changes introduced by researchers or computational techniques. These measures can not only improve the accuracy of reviews, but they can also contribute to researcher training by highlighting areas for improvement.

Third, data control further pertains to the integrity, availability, and confidentiality of literature review data. Integrity can be maintained by tracking who made what changes, using tools like *Git* for version control. Availability should allow for flexible deployment, whether locally or on servers, with the ability to roll back to any previous version for technical control. Confidentiality is also critical, especially when handling copyrighted PDFs, ensuring these materials are stored securely and accessed only by authorized review team members.

#### 3.4.3 Recommendations

*Select standard file formats to facilitate access and contribution by humans and software:* By adopting openly accessible and standardized file formats that are readable by both humans and software, authors of review papers can strategically enhance data transparency and tool interoperability. This requires not only choosing appropriate formats, but also actively exporting and maintaining review data outside proprietary tools so that it remains accessible for inspection, reuse, and automation. Proprietary software often restricts integration when data are not exposed through programmatic interfaces (APIs). In addition, formats, such as *enl*, *RIS*, or *CSV*, make it difficult to track and interpret changes, particularly in version-controlled workflows. In contrast, formats, such as *BibTeX*, offer distinct advantages due to their readability, version comparability, and compatibility with collaborative versioning systems.

*Adopt non-redundant data structures, supported by identifiers and automated validation procedures:* As highlighted by fundamental data management literature,^8^ minimizing redundant storage of data is integral to maintaining a consistent state of datasets. Attention to this recommendation appears particularly warranted in the context of literature reviews, where redundant data representations are relatively common. For instance, we note that the *SYNERGY* dataset has adopted a different structure, storing status information redundantly. This results in contradictory status values with raw data records associated with the same paper, as some records are marked as simultaneously included and excluded.^9^ Cases of contradictory values could be prevented by non-redundant data schemata. The same logic applies to aggregated outputs—such as statistics, visual paper profiles, or PRISMA diagrams—which should be derived programmatically from the current primary data instead of being stored redundantly.

*Assess all manual and computational changes and roll back unreliable contributions:* Authors play an active role in assessing the quality of input data and the reliability of any changes made during the literature review process, whether those changes are algorithmic, collaborative, or manual. Taking accountability for reviewing and selecting data contributions is critical to ensuring the integrity of the review. Additionally, authors must be aware of the limitations of current literature review tools, particularly in areas related to data control and auditability. As new tools emerge with improved tracking, versioning, and control features, authors should consider adopting these technologies to enhance the quality, reliability, and transparency of their reviews.

### 3.5 Consume: Use and reuse of literature review data

#### 3.5.1 State of the art and limitations

The publication of data for consumption in follow-up review projects is very limited, as shown by recent work.^82^ ^,^ ^83^ For instance, Page et al.^82^ found that data and code availability statements for systematic reviews were often missing or inaccurate. In fact, the most common statement related to data consumption was that data could be made available upon request. Especially in the management and organizational disciplines, there seems to be a lack of awareness of the different elements that could be made available, including the review protocol (e.g., on the *Open Science Framework* platform), the search strategy (e.g., on *searchRxiv*), and the screening decisions (e.g., in the *SYNERGY* datasets). These initiatives may see a broader uptake when more journals start to adopt open data policies.

Reusing data from other review projects when writing a new review article primarily relies on manual processes and is rarely supported by dedicated tools. Although several components of a review dataset could be leveraged in subsequent reviews, the challenges are particularly pronounced when attempting to reuse screening decisions from prior studies. Incorporating these screening decisions can serve as a valuable starting point to either broaden or narrow the scope of related review projects, helping to save time and reduce the risk of overlooking relevant studies. However, in the absence of adequate tool support, particularly for record linkage, limitations in data reuse hinder the efficiency and effectiveness of review practices.

#### 3.5.2 Principles

As a first principle, the reuse of literature review data presents a promising opportunity, focused on the ability to utilize curated review data across different projects, teams, and within the research community to enhance efficiency and quality. Despite the cumulative nature of research, the foundations for facilitating data reuse are currently underdeveloped, with no clear vision for automating this process. As a result, researchers often begin anew when exploring prior literature, even though curated content from previous reviews could be integrated to form background sections, update review papers, or conduct umbrella reviews. Reuse in literature reviews could apply to various steps, including integrating metadata and curated content or reusing screening decisions. We believe that—in contrast to primary empirical research—automated reuse is more important than replication in the context of literature reviews. Although some reuse occurs manually, it often remains inefficient and potentially error-prone. Initiatives, such as shared knowledge repositories and knowledge graphs, aim to address these challenges by enabling systematic reuse, contingent on the trustworthiness of the data sources.^71^ ^,^ ^84^ Furthermore, to advance reuse in literature reviews, it is crucial to make data available to evaluate and enhance existing tools and methodologies, like *SYNERGY* datasets for pre-screening algorithms.^78^

Second, the reuse of literature review data may occur either manually or algorithmically. To facilitate this, shared repositories should be designed for ease of access, enabling literature review data to be easily retrieved and seamlessly integrated into other research projects. This will not only allow researchers to build upon existing work more efficiently but also enhance the overall research workflow. Importantly, ensuring that the data are provided in machine-readable formats is essential. Prioritizing machine-readable formats will also contribute to the development and evaluation of tools that rely on structured data for advanced analyses.

#### 3.5.3 Recommendations

*Reuse data from prior literature review papers and curated repositories:* Authors should actively consider reusing existing review datasets, including search queries, screening decisions, and curated content, to reduce redundancy and improve efficiency in the review process. Accordingly, data publication should be treated as a core output of any review project. At a minimum, authors should make bibliographical records available; ideally, they should provide access to the entire project database, including extracted data, while complying with copyright restrictions. For empirical work, it is particularly important to offer machine-readable content that facilitates automated data extraction and seamless integration into new projects.

*Share review data via open data platforms and use open licenses to enable reuse:* Beyond individual projects, authors should consider how their data can contribute to broader review infrastructures, including protocol registries (e.g., *Open Science Framework*), search-query repositories (e.g., *searchRxiv*), and shared screening datasets (e.g., *SYNERGY*). For authors, it is essential to carefully select and organize data curation in review projects, to think beyond individual review projects, and envision how different reviewing efforts can be connected. Effective curation and open licenses are critical to allow key elements—such as queries, metadata, and inclusion decisions—to be recombined across studies. However, reuse depends not only on deposition but also on discoverability and interoperability. Initiatives like the *Cochrane API* ^10^ offer a useful model, illustrating how record-level and project-level content indexing can enable knowledge flows across projects, both within review teams and broader research communities. In the long run, such infrastructures can support continuous, program-level reviewing that transcends isolated projects while feeding individual syntheses as needed.

**Figure 4:**
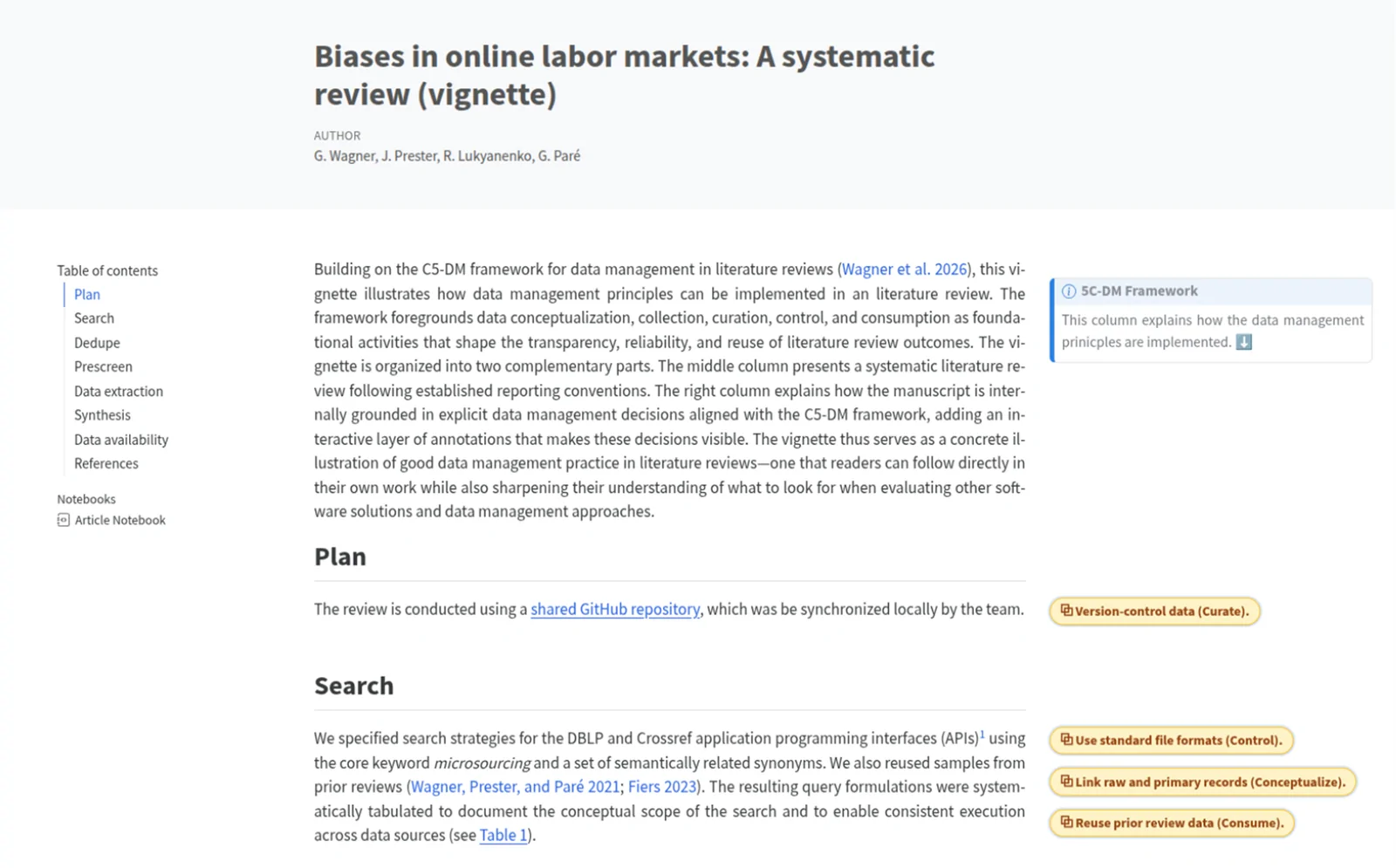
Online vignette with practical illustrations of the data management recommendations. *Note*: The interactive vignette is available at https://fs-ise.github.io/C5-DM-vignette/. Figure 4 Long description.

## 4 Tool development and evaluation

Drawing on data management principles and promoting collaboration between experts in review methodologies and tool developers is critical for driving innovation, enhancing transparency, and improving the efficiency of literature reviews. Each area of data management offers starting points for improvement.

With regard to *data conceptualization*, it is essential to advance the definition of data and data requirements. First, clarifying the data structures and, by extension, the corresponding review types supported by tools, is critical. Second, tools should provide transparent tracking of record statuses, disclosing and aligning the underlying models to improve interoperability and the tracking of records across the entire review process. Third, tools must anticipate collaborative editing by both humans and algorithms, incorporating mechanisms to ensure data integrity and to validate the results. Fourth, support for data transparency and provenance is crucial, offering clear visibility into how the review data evolve over time and enabling the ability to track changes and undo modifications when necessary.

In the *data collection* area, key issues remain to be addressed by tool designers and evaluators. The conundrum of updating literature searches remains to be addressed, despite the availability of fundamental data management techniques, such as unique identifier design and entity resolution.^56^ Efficient search updates should rely on transparent, reliable procedures that differentiate raw source data from integrated primary records while maintaining clear record lineage, schema integration, and data preparation procedures. Currently, most literature searches rely on manual retrieval through the web interfaces of academic databases.^52^ Automated, API-based searches are not yet widely supported, particularly for handling complex nested Boolean queries commonly used in standalone review papers. Advancements in design science are essential to realize the visions of open research synthesis^85^ and continuously updated reviews.^58^ Another research opportunity lies in leveraging LLM-based systems to streamline and enhance the literature collection process.

For the development of infrastructure to *curate review data*, we propose leveraging *Git* as a data management system due to its inherent capabilities for ensuring transparency, supporting validation processes, and enabling undo operations. This approach requires comprehensive efforts to design a robust conceptual model for *Git*-based literature reviews and to ensure data consistency throughout the lifecycle of review projects. In addition, mechanisms for record- and project-level data flows between review projects should be established through appropriate indexing and retrieval systems to facilitate seamless knowledge transfer.

With regard to the *control and provenance of data*, tool designers and evaluators should aim to establish a consensus on standardized data structures that can be implemented across various literature review tools. These structures should be non-redundant and inherently designed to prevent inconsistent states, thereby ensuring consistency by design. It is also essential for tools to incorporate audit trail functionality, enabling the tracking of all changes made to records. This capability allows researchers to analyze the quality of modifications introduced by both humans and computational techniques, thereby supporting accountability and transparency.

Concerning *data consumption*, there is a pressing need to support the publication of review datasets under licenses that explicitly allow for reuse. Tools should facilitate the publication, retrieval, and integration of curated content across different review projects. Additionally, open science platforms must be adapted to meet the specific needs of literature reviews, such as facilitating detailed record-level retrieval. By enhancing the accessibility, reusability, and traceability of literature review data, these improvements will make it easier for researchers to build upon existing work while maintaining the integrity of the underlying data.

More generally, tool developers can conceptualize literature review data as data that are fundamentally being reused or repurposed. Indeed, when researchers use metadata, abstracts, or past review results in new syntheses, they are engaging in repurposing. This means that creating the tools and infrastructure to support data management during literature reviews can benefit from advances in data management for repurposing,^86^ including paying more attention to the use-agnostic properties of data. The use-agnostic properties of data include its accessibility, transparency, and elasticity—these, in addition to other, traditional properties of data (e.g., accuracy and completeness) specifically promote data repurposing by optimizing the future and unanticipated data uses. For example, to enhance the elasticity of literature review data, it is recommended to ensure that all relevant literature review data are self-contained, that is, there is no need to consult other data sources to use these data. More generally, in developing the metrics to assess the quality of data for literature reviews, its use agnosticism, or repurpose-potential should be considered more closely.

## 5 Concluding remarks

This article is driven by the conviction that literature review tools and practices could benefit tremendously from a stronger foundation in data management. Although prior research has extensively engaged with the methodological procedures, the management of data remains relatively underdeveloped. As a result, review tools lag behind the integration of tools achieved in other types of scientific data analyses. Of the over 300 tools listed by *www.systematicreviewtools.com*, few are extensible, and none enable a seamless combination of extensions, as seen in the R and *tidyverse* environment.^3^ A key insight from this environment is that innovative and efficient recombination requires package extensions to “share an underlying design philosophy, grammar, and data structures.”^87^ In addition, researchers—in an effort to manage the enormous volumes of research available—increasingly turn to advanced computational techniques to support the conduct of literature reviews.^63^ ^,^ ^88^ It is critical that data management principles enable researchers to test, validate, and potentially undo changes from novel computational techniques.

The C5-DM Framework and its data management principles are aimed at helping researchers dedicate more time to critical analysis, prevent errors, and enrich the review process. Given the rapid growth of research output and increasing external demands, efficient tools for literature reviews, knowledge synthesis, and data reuse will be integral to fostering a strong cumulative tradition.

We offer actionable and specific recommendations to advance the conversation on open data and open science within the context of literature reviews. Addressing data management challenges is essential for advancing evidence synthesis communities,^85^ computational literature reviews,^89^ and living reviews.^90^ While individual actions can be taken within specific areas, we believe that the most significant benefits will emerge when efforts across different stakeholders and data management domains start to synergize. Progress in these areas depends on concerted efforts within the research community, with a focus on data standardization and tool innovation. Unlike data for statistical analyses, ML, or qualitative analyses, literature review data and tools have attracted limited interest from industry developers. Instead, experiences from fields, such as statistics, ML, and simulation studies, suggest that the active involvement of scientists and methodologists is essential for fostering innovation and meaningful progress at the intersection of data, tools, and research methods.

## Data Availability

Not applicable.

## Footnotes

1 The *SYNERGY* dataset is an open data collection of systematic reviews, primarily containing data on study selection.

2 As an example, see https://cookiecutter-data-science.drivendata.org/opinions/.

3 After *Google Scholar*, which has faced criticism for data quality issues,^47^ ^,^ ^48^ another prominent example is *Semantic Scholar*, known for its innovative application of natural language processing (NLP) and ML techniques. However, data quality does not appear to be a primary focus, as records frequently contain incomplete or inaccurate metadata. Given that LLM-based tools, such as *Elicit*, rely on *Semantic Scholar* as a data source, such errors can easily propagate into LLMs.

4 LLMs are large language models, a recent development in generative AI based on advanced context-aware deep learning neural networks.

5 Turing-complete programming languages, even relational databases, could, in principle, provide transparent change logs of unstructured data. But that would require extensive efforts, and we are not aware of any products in which developers actually attempted to adapt relational databases to the specific needs discussed earlier (see Section 3.1).

6 Initial projects explore *Git*-based review models, such as the COVID-19 review,^66^ which mostly covers the synthesis but not the systematic search and selection steps of a standalone literature review.

7 Different interpretations and paradigmatic lenses may even raise the need for layers of data, which may represent both complementary and conflicting views on the literature. An example of complementary layers is papers annotated for their research methods, citation relationships, or empirical findings. However, differences between interpretations may not always be eliminated, but figure as a valuable feature. For instance, interpretive reviews may follow the *principle of multiple interpretations*,^72^ and meta-narrative reviews are dedicated to understanding how different paradigms lead to seemingly contradictory theoretical solutions, without forcing a “simple, formulaic, or universal ‘solution’ ” upon the literature.^73^

8 At this point, we refer to transparency at the level of data that is available internally, during the conduct of the review. This does not necessarily coincide with external transparency, or the details reported in the article.^79^

9 For example, in the dataset https://github.com/asreview/synergy-dataset/blob/master/datasets/Appenzeller-Herzog_2019/Appenzeller-Herzog_2019_ids.csv, for the first five raw data records, there are other raw data records referring to the same paper with contradictory status information (label_included = 1 vs. 0).

10 See https://test-api.cochrane.org/api-docs/index.html.

## Long descriptions

### Table 1 Long description

The table consists of four columns and eight rows of data.

* Row 1: P R I S M A 2020 and extensions. Primary purpose is reporting of systematic reviews. Provides standards for reporting search, screening, and synthesis. Does not provide guidance on data structures, versioning, or workflows.

* Row 2: E N T R E Q. Primary purpose is reporting of qualitative syntheses. Provides standards for reporting qualitative methods and synthesis. Does not provide guidance on coding files, extraction structure, and traceability.

* Row 3: R A M E S E S I and I I. Primary purpose is method and reporting of realist reviews. Provides protocol, synthesis logic, and reporting standards. Does not provide data formats, versioning, or interoperability.

* Row 4: A M S T A R I and 2. Primary purpose is critical appraisal of systematic reviews. Provides criteria to evaluate methodological rigor. Does not provide guidance on data preparation, screening workflows, and schemata.

* Row 5: G R A D E. Primary purpose is evaluation of certainty of evidence. Provides rules for rating evidence certainty. Does not provide data formats, extraction structures, or literature review processes.

* Row 6: F A I R. Primary purpose is principles for scientific data stewardship. Provides principles related to findability, accessibility, interoperability, and reusability. Does not provide literature review-specific schemata or data lifecycle guidance.

* Row 7: P I C O S or S P I D E R. Primary purpose is structure for review questions and search strategies. Provides templates for defining inclusion criteria and creating search strategy building blocks. Does not provide guidance on data inputs, logs, extraction, or transformations.

* Row 8: C 5-D M (this article). Primary purpose is data lifecycle management for literature reviews. Provides principles and practical recommendations for managing literature review data. Does not replace standards for reporting or evidence assessment.

Navigate back to Table 1.

### Table 2 Long description

The table is organized into two columns: Areas and principles, and Data management recommendations.

1. Conceptualize: Principles state that literature review data covers input, process, and output, and is characterized by dynamics and atomicity. Recommendations include storing search results separately from primary data and documenting manual work as primary analytical procedures.

2. Collect: Principles emphasize defining metadata quality and the benefits of A P I-based searches over manual interfaces. Recommendations include documenting database coverage for L L M tools and setting up automated data preparation.

3. Curate: Principles highlight the need for transparent systems and fitting data structures to the review type. Recommendations include using version-controlled repositories and aligning structures with specific review types.

4. Control: Principles focus on standardized structures for interoperability and quality management. Recommendations include selecting standard file formats, adopting non-redundant structures, and assessing all changes for rollback if needed.

5. Consume: Principles define reuse as the use of metadata or screening decisions in follow-up research. Recommendations include reusing data from prior papers and sharing data via open platforms with open licenses.

Navigate back to Table 2.

### Figure 1 Long description

The diagram is divided into four horizontal sections from top to bottom.

1. Planning: Contains a box for Project setup including search strategy, screening criteria, and team members.

2. Input: Features two main components. Record (raw data) includes metadata and identifiers. Full-text document includes P D F, website, and appendices.

3. Review process (iterative): This central section shows the flow between four boxes.

- Primary record (metadata, identifiers list, status) points to Full-text document with the label represents.

- Extracted data (evidence, concepts, themes, statistics) points to Full-text document with the label synthesizes.

- Changes (manual, collaborative, computational) has a dashed arrow pointing to Primary record.

- An Errors label with squiggly arrows points toward the connections between Changes, Primary record, and Extracted data.

- Primary record, Full-text document, and Extracted data each feature a circular loop arrow indicating iteration.

4. Output: The final stage contains two boxes. Review dataset includes sample and screening decisions. Manuscript includes synthesis, methods report, references, P R I S M A diagram, and profile of papers.

Navigate back to Figure 1.

### Figure 2 Long description

The workflow consists of three main stages from left to right.

1. Left Stage: Raw data record(s). Represented by a stack of three rectangular boxes. A label above states: Search results stored separately and in the original data format (e.g., R I S, C S V). Inside the front box, bullet points list Identifiers and Metadata.

2. Center Stage: Transformation. Three horizontal arrows point from the raw data records toward the right. Above the arrows, text reads: Converted into a common and expressive format (e.g., BibTeX). Below the arrows, text reads: Original raw data records are linked to master records (when merging duplicates).

3. Right Stage: Primary record. A single large rectangular box. A label above states: Assigned to unique and non-modifiable I D. Inside the box, bullet points list Identifiers, Origin links, Status, and Metadata. A label below the box states: Progress tracked based on a status field (e.g., included, data_extracted).

Navigate back to Figure 2.

### Figure 3 Long description

A horizontal double-headed arrow at the bottom serves as a scale, labeled Structured on the left and Unstructured on the right. Above this axis are four distinct data representations.

1. Tabular (Far Left): A grid table with three columns and five rows. The first column contains bracketed numbers [1] through [4]. The headers and cell contents are blurred placeholder text.

2. Emergent (Top Center): A flowchart-style diagram. Four rectangular boxes on the left contain blurred text and bracketed numbers [1], [4], [2], and [1]. Lines connect the top two boxes to a single box labeled Dolor sit, and the bottom two boxes to a box labeled Elitr.

3. Graph-based (Bottom Center): A network diagram consisting of three large circles labeled [1], [2], and [3], and three smaller unlabeled circles. All circles are interconnected by a complex web of straight lines.

4. Narrative (Far Right): A block of blurred justified text. Within the text, bracketed citations [1], [3,4], and [2] are visible, representing data embedded within a natural language format.

Navigate back to Figure 3.

### Figure 4 Long description

The layout is divided into three distinct vertical zones.

* Left Sidebar: Contains a Table of contents with links to Plan, Search, Dedupe, Prescreen, Data extraction, Synthesis, Data availability, and References. Below this is a Notebooks section with a link to an Article Notebook.

* Central Column: Titled Biases in online labor markets A systematic review vignette by G. Wagner, J. Prester, R. Lukyanenko, and G. Paré. It contains introductory text explaining the C 5 D M framework for data management. Below the introduction are two sections: Plan, which mentions a shared GitHub repository, and Search, which describes search strategies for D B L P and Crossref A P I s.

* Right Sidebar: Features interactive annotations. At the top is a blue box labeled 5 C D M Framework explaining how principles are implemented. Further down, yellow pill-shaped buttons align with the central text, including Version-control data (Curate), Use standard file formats (Control), Link raw and primary records (Conceptualize), and Reuse prior review data (Consume).

Navigate back to Figure 4.
